# *N*-Acetylcysteine and a Specialized Preventive Intervention for Individuals at High Risk for Psychosis: A Randomized Double-Blind Multicenter Trial

**DOI:** 10.1093/schizbullopen/sgae005

**Published:** 2024-02-28

**Authors:** Sven Wasserthal, Ana Muthesius, René Hurlemann, Stephan Ruhrmann, Stefanie J Schmidt, Martin Hellmich, Frauke Schultze-Lutter, Joachim Klosterkötter, Hendrik Müller, Andreas Meyer-Lindenberg, Timm B Poeppl, Henrik Walter, Dusan Hirjak, Nikolaos Koutsouleris, Andreas J Fallgatter, Andreas Bechdolf, Anke Brockhaus-Dumke, Christoph Mulert, Alexandra Philipsen, Joseph Kambeitz

**Affiliations:** Division of Medical Psychology, Department of Psychiatry and Psychotherapy, University Hospital of Bonn, Bonn, Germany; Department of Psychiatry and Psychotherapy, University of Cologne and University Hospital Cologne, Cologne, Germany; Department of Psychiatry, School of Medicine and Health Sciences, University of Oldenburg, Oldenburg, Germany; Department of Psychiatry and Psychotherapy, University of Cologne and University Hospital Cologne, Cologne, Germany; Division of Clinical Child and Adolescent Psychology, University of Bern, Bern, Switzerland; Faculty of Medicine and University Hospital Cologne, Institute of Medical Statistics and Computational Biology, University of Cologne, Cologne, Germany; Department of Psychiatry and Psychotherapy, Medical Faculty, Heinrich-Heine University, Düsseldorf, Germany; Department of Psychology, Faculty of Psychology, Airlangga University, Surabaya, Indonesia; University Hospital of Child and Adolescent Psychiatry and Psychotherapy, University of Bern, Bern, Switzerland; Department of Psychiatry and Psychotherapy, University of Cologne and University Hospital Cologne, Cologne, Germany; Department of Psychiatry and Psychotherapy, University of Cologne and University Hospital Cologne, Cologne, Germany; Department of Psychiatry and Psychotherapy, Central Institute of Mental Health, University of Heidelberg/Medical Faculty Mannheim, Mannheim, Germany; Department of Psychiatry and Psychotherapy, University of Regensburg, Regensburg, Germany; Department of Psychiatry, Psychotherapy and Psychosomatics, RWTH Aachen University, Aachen, Germany; Department of Psychiatry and Psychotherapy CCM, Charité, Universitätsmedizin Berlin, Corporate Member of Freie Universität Berlin, Humboldt-Universität zu Berlin, and Berlin Institute of Health, Berlin, Germany; Department of Psychiatry and Psychotherapy, Central Institute of Mental Health, University of Heidelberg/Medical Faculty Mannheim, Mannheim, Germany; Department of Psychiatry and Psychotherapy, Ludwig Maximilian University of Munich, Munich, Germany; Department of Psychiatry and Psychotherapy, Tübingen Center for Mental Health, University of Tübingen, Tübingen, Germany; German Center for Mental Health (DZPG), Partner Site Tübingen, Tübingen, Germany; Department of Psychiatry and Psychotherapy CCM, Charité, Universitätsmedizin Berlin, Corporate Member of Freie Universität Berlin, Humboldt-Universität zu Berlin, and Berlin Institute of Health, Berlin, Germany; Department of Psychiatry, Psychotherapy and Psychosomatic Medicine with Early Intervention and Recognition Center (FRITZ), Vivantes Klinikum Am Urban, Berlin, Germany; Department of Psychiatry and Psychotherapy, LVR-Clinic Bonn, Bonn, Germany; Center of Psychiatry, Justus-Liebig University, Giessen, Germany; Department of Psychiatry and Psychotherapy, University Hospital of Bonn, Bonn, Germany; Department of Psychiatry and Psychotherapy, University of Cologne and University Hospital Cologne, Cologne, Germany

**Keywords:** *N*-acetylcysteine, clinical high risk, integrated intervention, social functioning

## Abstract

**Background and Hypothesis:**

Clinical high risk for psychosis (CHR-P) offers a window of opportunity for early intervention and recent trials have shown promising results for the use of *N*-acetylcysteine (NAC) in schizophrenia. Moreover, integrated preventive psychological intervention (IPPI), applies social-cognitive remediation to aid in preventing the transition to the psychosis of CHR-P patients.

**Study Design:**

In this double-blind, randomized, controlled multicenter trial, a 2 × 2 factorial design was applied to investigate the effects of NAC compared to placebo (PLC) and IPPI compared to psychological stress management (PSM). The primary endpoint was the transition to psychosis or deterioration of CHR-P symptoms after 18 months.

**Study Results:**

While insufficient recruitment led to early trial termination, a total of 48 participants were included in the study. Patients receiving NAC showed numerically higher estimates of event-free survival probability (IPPI + NAC: 72.7 ± 13.4%, PSM + NAC: 72.7 ± 13.4%) as compared to patients receiving PLC (IPPI + PLC: 56.1 ± 15.3%, PSM + PLC: 39.0 ± 17.4%). However, a log-rank chi-square test in Kaplan–Meier analysis revealed no significant difference of survival probability for NAC vs control (point hazard ratio: 0.879, 95% CI 0.281–2.756) or IPPI vs control (point hazard ratio: 0.827, 95% CI 0.295–2.314). The number of adverse events (AE) did not differ significantly between the four groups.

**Conclusions:**

The superiority of NAC or IPPI in preventing psychosis in patients with CHR-P compared to controls could not be statistically validated in this trial. However, results indicate a consistent pattern that warrants further testing of NAC as a promising and well-tolerated intervention for CHR patients in future trials with adequate statistical power.

## Introduction

Psychotic disorders rank high on the global burden of disease statistic^1^ and are often associated with a considerable loss of psychosocial function and quality of life.^2^ Early detection and prevention aim to delay or even prevent transition to psychosis and functional decline. While clinical criteria for the detection of high risk for psychosis are well established^3^ and offer a window of opportunity for early intervention almost unique in psychiatry,^3,4^ there is an urgent need for the development of effective and tolerable interventions that facilitate the implementation of early intervention approaches.

The administration of second-generation antipsychotic substances in patients with clinical high risk for psychosis (CHR-P) has been shown to reduce symptom load in clinical trials.^5^ However, antipsychotics have a significant risk of causing unfavorable side effects. Furthermore, over the last 10 years, a steady overall decline in transition rates of CHR-P patients has been observed in various studies^6^ and only about one-fifth of CHR-P patients experience transition to psychosis within 2 years.^6^ Even though mixed results on the efficacy of neuroprotective and anti-inflammatory agents like omega-3-fatty acids, d-serine and cannabidiol^7–10^ were obtained,^10–12^ aggregation of the available evidence in meta-analyses showed benefits for various experimental interventions.^3,13,14^

In this context, *N*-acetylcysteine (NAC) provides an intriguing pathway for potential treatment in CHR-P. The neuroprotective effects of NAC are mediated by three distinct mechanisms^15^: (1) Mitigation of oxidative stress through cysteine donation; (2) decrease of neuro-inflammation by attenuating cytokine levels; and (3) modulation of glutamatergic signaling by activating the cysteine-glutamate antiporter. All three pathways have been shown to be involved in the pathophysiology of schizophrenia on several occasions.^16–19^ Glutamatergic signaling can also be manipulated using NMDA-receptor antagonists like ketamine.^20^ Subanesthetic ketamine induces psychotomimetic states in humans and rodents similar to schizophrenia.^21^ Interestingly, perinatal ketamine treatment and subsequent NAC application in mice prevented the development of cognitive and social behavioral deficits.^22^ Additionally, a transgenic mouse model with a glutathione deficit showed recovery of oxidative damage by applying NAC.^23^

The compound was also shown to improve mismatch negativity,^24^ processing speed,^25^ and working memory^26^ in patients with schizophrenia. In chronic schizophrenia, improvement of negative symptoms and neurocognitive functioning were demonstrated.^27^ For individuals with CHR-P, clinical trials demonstrated that (1) NAC supplementation increases glutathione levels, (2) has a positive effect on functional connectivity within the cingulate cortex,^28^ and (3) improves negative and disorganized symptoms.^29^ Due to its assumed neuroprotective nature and positive effects on cognition and symptoms, NAC is thus a promising agent in the prevention of psychosis. A case report with five CHR-P patients found a potential benefit for the treatment.^30^

Psychological treatments also meet the criterion of a low side-effects profile and are generally recommended as the first-line treatment of CHR-P.^3^ Psychological interventions for CHR-P that have been investigated in randomized controlled trials are cognitive behavioral therapy (CBT),^31^ integrated psychological treatment,^32^ and family therapy.^33^ While all interventions showed generally favorable effects, no specific intervention was superior in preventing psychosis in CHR-P patients so far.

Several studies indicated that social functioning is a crucial target for preventive approaches.^34^ It is predictive for transition to psychosis, impaired in CHR-P states, and persists even after remission of CHR-P symptoms.^35–37^ Generally, the effects of various cognitive behavioral therapies on social functioning were shown to be rather small.^38^ However, in a cohort of youth with CHR-P, a remediation intervention was recently shown to have favorable effects on mentalizing.^39^ Integrated preventive psychological intervention (IPPI) is a novel psychotherapeutic intervention to provide disorder-related knowledge, improve social functioning, and stress/symptom management, and applies social-cognitive remediation.^40^

The aim of this study was to investigate individual and combined effects of the two different interventions (NAC or IPPI vs Placebo or PSM) on the transition to psychosis within CHR-P patients by focusing on amelioration of glutamatergic signaling with NAC, symptom management, and improving social cognition with IPPI. The application of both interventions in combination with a control-condition or in combination with each other, aimed to study their individual as well as their combined effects simultaneously. We hypothesized that treatment groups receiving both treatments (NAC and IPPI), would show significantly fewer transitions to psychosis, less deterioration of CHR-P symptoms (primary outcome), and improved social functioning, social cognition, and neurocognitive capabilities (secondary outcome) compared to patients in one or both placebo groups.

## Methods

### Participants

Between 2016 and 2021, eleven German trial sites recruited 48 subjects in this double-blind (single-blind for psychotherapeutic intervention) placebo-controlled, randomized clinical trial. Participants were recruited via the center’s early detection facilities and either self-referred or referred via practitioners in stationary or ambulant settings. Inclusion criteria were (1) fulfilling criteria for CHR-P as assessed by the Structured Interview for Psychosis-Risk Syndromes (SIPS)^41^ and the Schizophrenia Proneness Instrument, Adult version (SPI-A)^42^ and (2) decreased social functioning as measured with the Social and Occupational Functioning Assessment Scale^43^ (SOFAS) and the Global Assessment of Functioning^44^ (GAF). Exclusion criteria were, among others, a past psychotic episode spanning more than 7 days, lifetime antipsychotic medication with a cumulative dosage of over 30 times the minimum effective dose according to S3-Guidelines for schizophrenia, and any past psychotherapeutic training for prevention purposes. Further details on inclusion and exclusion criteria, as well as trial design and recruitment, can be found in Schmidt et al^40^ and in Supplementary table 1. A CONSORT chart is available in the supplement.

### Trial Design

**Fig. 1.**
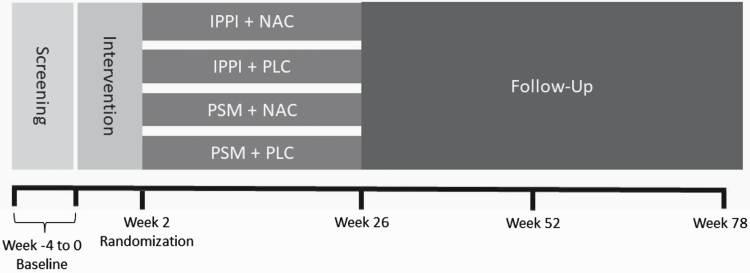
Study design: the trial comprises a 2 × 2 factorial design with four study arms. The intervention spans 26 weeks with a follow-up period of up to 52 weeks. IPPI, integrated preventive psychological intervention; NAC, *N*-acetylcysteine; PSM, psychological stress management; PLC, placebo.

The trial features a 2 × 2 factorial design with four arms to assess combined and single effects of NAC vs Placebo (PLC) and integrated preventive psychological intervention (IPPI) vs psychological stress management (PSM) (see figure 1). PSM is believed to enhance coping mechanisms and stress management among patients grappling with psychotic symptoms, potentially contributing to a reduction in the severity of these symptoms.^45^ It was selected as the active control-condition for the psychological intervention, aiming to discern the specific impact of enhanced social cognition on symptoms in individuals at risk for psychosis presented only in the IPPI sessions. The intervention period spanned 26 weeks, with a follow-up period of up to 52 weeks. Randomization to one of four arms was done stratified by trial center via an internet service (ALEA; FormsVisionBV, Abcoude, NL; https://www.aleaclinical.eu/) and took place after obtaining informed consent and a baseline visit. For randomization, blocks of varied lengths were permuted to create allocation sequences. Results of the randomization were displayed on screen and communicated to approved staff members through e-mail. Follow-up assessments took place at weeks 13, 26, 52, and 78. Raters remained blinded to all conditions, as IPPI and PSM were carried out by trained therapists. To this end, generated data from psychotherapeutic sessions was kept separate from data obtained by raters in bi-weekly visits.

### Interventions and Questionnaires

NAC (Hexal, Holzkirchen, Germany) or PLC were provided as two capsules containing 500 mg of the compound two times a day, amounting to 2000 mg/day. Mode of ingestion and dosage were chosen in accordance with earlier research^27^ demonstrating safety, tolerability, and good bioavailability.^46^ Capsules were manufactured and provided by the pharmacy of the University Hospital in Heidelberg. PLC capsules contained a filling agent (mannitol and aerosil), frequently used for medical trials.

IPPI was developed with the goal of preventing a transition to psychosis by improving stress management, symptom management as well as social cognition. This manualized therapy is comprised of 21 weekly sessions and a final booster session, and each module focuses on motivation using multi-sensory materials in social cognition domains (Theory of Mind and empathy, affect recognition, social perception, social attributions, and social problem solving) as well as symptom and stress management—further details are described in Schmidt et al.^40^ A psychological stress management (PSM) intervention was introduced as an unspecific control-condition and spanned across 11 bi-weekly sessions and a final closing-session. It aims at improving coping with stressful situations in patients leaning on the vulnerability-stress-coping model of the development of psychosis.^45,47–49^ Psychotherapists with at least advanced postgraduate training conducted both IPPI and PSM, ensuring their professional adherence to the highly manualized protocols. Throughout the trial period, therapists had the option to seek supervision from SJS at any time. Additionally, therapists received supervision during monthly meetings that involved participating therapists from all centers. Co-primary outcome variables assessing social functioning were operationalized by the Social and Occupational Functioning Assessment Scale (SOFAS) and Functional Remission of General Schizophrenia (FROGS) questionnaire. While the FROGS contains five subscales (daily life, activities, relationships, quality of adaption, and health and treatments), the SOFAS consists of a single scale ranging from low social functioning (score of 0) to perfect functioning (score of 100). A significant change from baseline in either instrument indicated improvement or worsening of social functioning. Secondary variables were quantitative changes in scores of neurocognitive assessments, ie, Digit Symbol Substitution Test^50^ (DSST), Verbal Learning and Memory Test^51^ (VLMT), Digit Span,^50^ Trail Making Test Versions A + B^52^ (TMT); improvement of negative and disorganization symptoms assessed by the Brief Negative Symptom Scale^53^ (BNSS) and SIPS; remission of CHR-P-criteria, depressive symptoms in the Calgary Depression Scale for Schizophrenia^54^ (CDSS), and social cognition assessed by the Movie for the Assessment of Social Cognition^55^ (MASC), the Social Attribution Test Multiple Choice^56^ (SAT-MC), and the Pictures of Facial Affect^57^ (PFA). Further secondary outcomes were the occurrence of adverse events (AE),^58^ adherence assessed with the Drug Attitude Inventory^59^ (DAI) and the Patient Questionnaire on Therapy Expectations and Evaluation (PATHEV), subjective quality of life according to the WHO-Quality-of-life Questionnaire (WHO-QOL^60^), laboratory assessments and body weight from baseline over time. A comprehensive overview of all outcome variables and their operationalization is available in Supplementary table 2.

### Statistical Analysis

Originally, a transition risk of 22% within 18 months had been assumed. During recruitment, new research^61^ led us to assume a transition risk of about 30% within the same timeframe for patients with impaired social and role functioning, as measured with the GAF. Since the probability of transition increased when impaired social functioning was introduced as an inclusion criterion (see Supplementary table 1), less patients per group were required to measure primary and secondary outcomes. To detect a relative reduction in transition risk of 80%, at a two-sided level of 2.5%, an uncorrected chi-square test would have required 48 patients to be recruited per group (IPPI/NAC; IPPI/PLC; PSM/NAC; PSM/PLC). To compensate for the influence of about 25% drop-out, it was planned to include *n* = 32 patients per study group. This resulted in *n* = 128 patients as the adjusted aim for the trial, with 32 patients per study arm. A futility analysis was performed in January 2020. The Data Safety Monitoring Board decided to terminate the trial prematurely, as the conditional power for the primary analysis was below 80% due to a lower number of eligible patients than anticipated during the specified time frame.

Primary analysis was based on the full analysis set, as derived from the intention-to-treat (ITT) principle. All randomized patients were included. Prior to this analysis, patient data was reviewed in a blind manner to determine evaluability. Patients who withdrew or showed protocol violations were included in the ITT population. One patient was accidentally unblinded, as they received a wrong medication kit due to an error in the randomization software and were consequently dropped from the study. Data of dropouts was analyzed using all available data. The primary outcome variable is the time from randomization to transition to psychosis or deterioration of symptoms defined by SPI-A and SIPS within up to 18 months. Based on the assumed progressive temporal link of symptom complexes “cognitive disabilities” (COGDIS), “attenuated psychotic symptoms” (APS), and “brief limited intermittent psychotic symptoms” (BLIPS),^62^ deterioration was defined as (1) fulfilling the diagnostic criteria for APS if COGDIS had been present before and (2) fulfilling the criteria of BLIPS if APS had been present before. The inclusion of symptom deterioration to the primary endpoint was deemed important due to the relatively truncated follow-up period of up to 12 months, which falls short of the average duration required for transition in the CHR-P demographic.^6^ Transition to psychosis was defined as the presence of at least one SIPS-positive symptom with a severity score of 6 (“severe and psychotic”) for >7 days. The comparisons of IPPI vs PSM and NAC vs PLC were based on stratified (by center) Cox-regression with main effects IPPI/PSM and NAC/PLC. Centers with fewer patients were pooled for this analysis. In this model, transitions and deterioration were defined as events within a survival analysis. As an estimate of effect size, hazard ratios were expressed in percentage of intervention groups showing event-free survival. Possible interactions were explored in the regression model. The proportional hazards assumption was explored by examining Kaplan–Meier plots and tested by introducing time-dependent covariates.

High censoring in data, leading to possible selection bias, was adjusted with inverse probability weighted (IPW) estimation.^63^ Inverse probability weights were used to create a pseudopopulation that is random with regard to the measured determinants of loss to follow-up, applying adjusted weights to each participant not lost to follow-up. These weights were then imputed into stratified (by center) cox-regression with covariates age and sex.

Both, co-primary and secondary endpoints were analyzed using mixed models for repeated measures with corresponding contrasts (assuming sufficient approximation by normal distributions, supported by visual inspection of the data) or using generalized estimating equations to describe and evaluate differences between groups and changes over time. Cohen’s *d* was calculated as effect size for visits at week 12, 26, and 78 and then averaged across visits. Data were analyzed with SPSS version 26 (IBM Corp., Armonk, NY, USA) and SAS.

### Safety and Tolerability

Adverse events were mainly specified by (1) items on the Udvalg for Kliniske Undersogelser side effect rating scale (UKU-SERS),^58^ which explores different domains of functioning within psychopharmacology, and (2) abnormal laboratory values.

The trial protocol was approved by the local ethics committees of lead centers Bonn and Cologne and subsequently approved by all ethics departments of participating trial sites. It was registered as Phase III trial with the Federal Institute for Drugs and Medical Devices and is registered with clinicaltrials.gov (NCT03149107) and European Eudra-CT (2014-003076-22). It was carried out in compliance with the Good Clinical Practice guidelines of the Declaration of Helsinki. The trial was sponsored by the Federal Ministry of Education and Research (Grant/Award Number: 01EE1407C, 01EE14071).

## Results

### Recruitment and Demographics

49 Participants were recruited, informed, and consent was obtained. 48 Patients were randomly assigned to one treatment group (NAC + IPPI, NAC + PSM, PLC + IPPI, PLC + PSM) after a baseline-visit. One patient terminated study participation before randomization due to the prescription of antipsychotic medication. In total, 23 patients received NAC and 24 patients participated in IPPI (for details, see Supplementary table 3). A total of 32 patients dropped out of the study. Of these, 23 dropped out during the intervention period. The most frequent reason named for drop-out was “termination by patient” (*n* = 9), followed by “loss to follow-up” (*n* = 3), and “protocol violations” (*n* = 3).

A Kruskal–Wallis test revealed no relevant differences between treatment groups in key demographic factors, even though age [range group means: 20.9 (PSM + PLC)—27.1 (NAC + PSM); *P* = .016] and urbanization [range small towns (<5.000): 0 (NAC + IPPI/NAC + PSM)—5 (PSM + PLC); range big cities (>1.000.000): 1 (PSM + PLC)—8 (IPPI + PLC/PSM + NAC); *P* = .015] showed statistical significance before multiplicity correction (ie, according to Bonferroni, see Supplementary table 3).

### Primary Endpoints

Intention-to-treat Kaplan–Meier analysis of the primary outcome “transition to psychosis” revealed 16 events (transition to psychosis) (*n* = 46, 30 censored times) at the end of the maximum follow-up period of up to 78 weeks. The overall median time-to-event was 43.0 weeks (*SE* = 9.6 weeks). For the primary endpoints data is presented as the rate of event-free survival, showing percentages of patients that did not transition to psychosis.

**Fig. 2.**
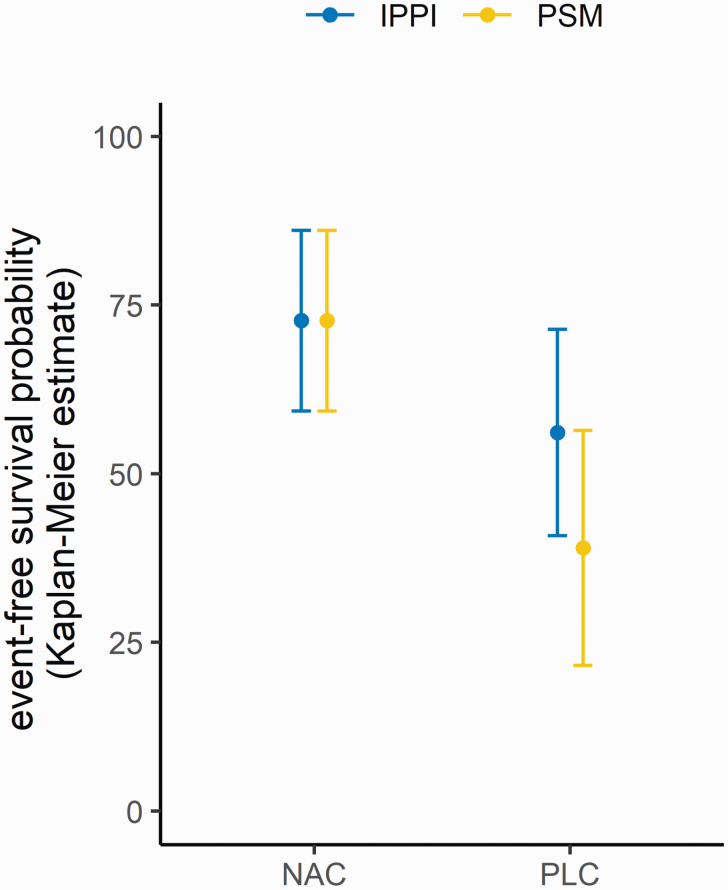
Estimates of event-free survival probability as derived from Kaplan–Meier analysis indicating lower probability of transition to psychosis in patients receiving *N*-acetylcysteine as compared to patients receiving placebo. NAC, *N*-acetylcysteine; IPPI, integrated preventive psychological intervention; PLC, placebo; PSM, supportive counseling.

**Fig. 3.**
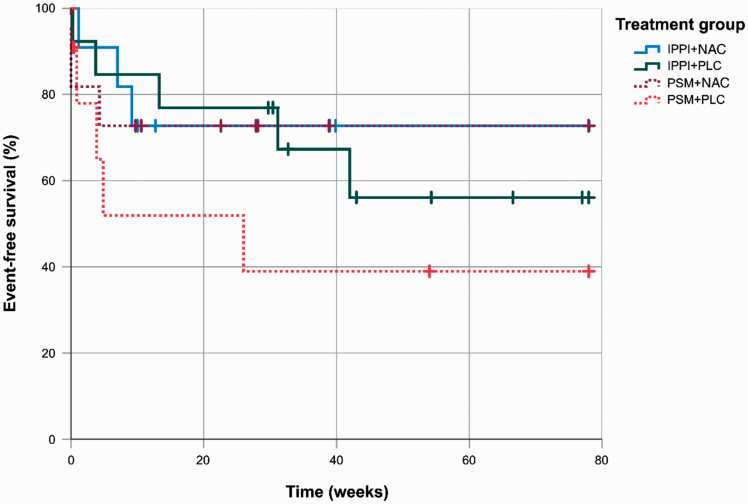
Kaplan–Meier survival analysis showing a tendency for higher survival probability within NAC treatment-groups. NAC, *N*-acetylcysteine; IPPI, integrated preventive psychological intervention; PLC, placebo; PSM, supportive counseling.

Overall event-free survival for IPPI was 62.3 ± 11.0% after 18 months, while this probability for the control-condition (PSM) was 57.6 ± 11.8% (*P* = .398, log-rank test; hazard ratio IPPI vs PSM 0.827, 95% CI 0.295–2.314). For NAC, the total event-free survival probability was 73.0 ± 9.4%, with its control-condition presenting at 50.5 ± 11.4% (*P* = .333; hazard ratio NAC vs PLC 0.879, 95% CI 0.281–2.756). Event-free survival probability after 18 months for the combined interventions was 72.7 ± 13.4% for NAC + IPPI (*P* = .674, hazard ratio vs PLC + PSM 0.707, 95% CI 0.141–3.549), 72.7 ± 13.4% for NAC + PSM (*P* = .730, hazard ratio vs PLC + PSM 0.785, 95% CI 0.197–3.119), 56.1 ± 15.3% for PLC + IPPI (*P* = .814, hazard ratio vs PLC + PSM 0.815, 95% CI 0.149–4.457), and 39.0 ± 17.4% for PLC + PSM (*P* = .504, overall log-rank test, see figures 2 and 3). In summary, no statistically significant difference between the transition rates of the intervention groups was found.

To adjust for possible selection bias due to high censoring, inverse probability weighting was used for stratified (by center) cox-regression with covariates age and sex.^63^ Inverse probability weighted time-to-event curves appeared congruent to unweighted (conventional) time-to-event (Kaplan–Meier) curves upon visual inspection, indicating that bias due to informative censoring may be negligible. In an exploratory analysis, (1) the effect of sex was as expected (male vs female HR = 0.778, 95% CI 0.248–2.439, *P* = .667) albeit not statistically significant, (2) the influence of center (pooled Wald-test = 1.110 with 3°C of freedom, *P* = .775) appeared unobtrusive, and (3) the effect of compliance, defined as having attended at least 80% of all expected therapy sessions or having taken at least 80% of medication provided, was again as expected (HR 0.405, 95% CI 0.137–1.196, *P* = .102), however, not statistically significant, either.

### Co-primary Endpoints

To calculate co-primary endpoints, a model with main effects for both treatments (compound and psychotherapy), using the baseline value as a covariate, was fitted. Then, an interaction between both treatments was added. No main effects or interactions yielded significant results for social functioning [FROGS: *F*(1, 27.88) = 0.01, *P* = .909; SOFAS: *F*(1, 27.97) = 0.50, *P* = .485].

### Secondary Endpoints

In total, 95 AEs were recorded. A majority of the recorded AEs were items on the UKU-SERS, used to assess different possible side effects in patients. The most frequent AE were abnormal dreams (*n* = 7), disturbance in attention (*n* = 6), tension (*n* = 5), and memory impairment (*n* = 4). Any other AEs were named a maximum of three times (see Supplementary table 4). The most frequent organ system class were psychiatric disorders (*n* = 28), nervous system (*n* = 13), and gastrointestinal system (*n* = 10). Three serious AE leading to hospitalization of the patient were reported. Reasons stated for hospitalization were “acute stress disorder” (*n* = 2) and “prodromal stage” (*n* = 1). None of the stated SAE were defined as having a certain or probable causal relationship to any of the applied treatments. Seven (S)AEs were classified as of “moderate” intensity, the rest as “mild.”

Pairwise comparisons between groups (NAC vs PLC and IPPI vs PSM) of different types of AE did not yield significant differences in frequency. A one-factorial Analysis of Variance (ANOVA) did not show any significant differences between the groups (NAC + IPPI vs PLC + IPPI vs NAC + PSM vs PLC + PSM: *F*(3, 42) = 0.70, *P* = .560), pointing towards good tolerability of the compound.

### Psychopathological and Psychological Measures

To assess the effect of treatments on different domains, every score was fed into a mixed model ANOVA with and without interaction (see Supplementary tables 5 and 6).

While no statistically significant differences were identified, interactions (group*visit) in mixed models showed tendencies towards differences between groups (IPPI vs PSM) for the BNSS reaction scale [*F*(2, 9.60) = 3.98, *P* = .055, *d* = 0.09], leaning towards stronger remission of lacking emotional reactions to stressful events in participants receiving psychotherapeutic treatment. Similarly, the total CDSS value showed a tendency for greater reduction in participants receiving IPPI [*F*(1, 95.28) = 3.43, *P* = 0.067, *d* = 0.09], indicating a stronger decline of depressive symptoms. However, participants of the control group (PSM + PLC) showed a shift towards stronger improvement in the WHO-QOL environment scale [*F*(2, 17.88) = 3.56, *P* = .050, *d* = 0.26]. This scale measures the quality of the physical environment surrounding the patient. Lastly, group differences between NAC vs PLC showed a tendency for significant interaction in the PATHEV hopefulness scale [*F*(2, 31.46) = 0.54, *P* = .041, *d* = 0.30], showing higher increments of hopefulness about the future in the PLC group. When Bonferroni correction for multiple testing was applied (*n* = 36), the critical *P*-value for all measures was reduced to *P*_crit_ .0014.

Lastly, we examined non-significant psychological measures whose effect sizes exceeded *d* = 0.50 (medium effect size) and did not exhibit floor effects and compared the outcomes between the contrasts IPPI vs PSM and NAC vs PLC. Our results showed that patients in the IPPI group demonstrated higher scores in SAT-MC II [*F*(2, 23.92) = 0.52, *P* = .476, *d* = 0.63] and PFA [*F*(2, 11.84) = 0.25, *P* = .780, *d* = 0.82], which are indicative of better social functioning. Interestingly, the alogy [*F*(2, 13.08) = 0.95, *P* = .410, *d* = 0.55] and avolition [*F*(2, 6.07) = 0.89, *P* = .457, *d* = 0.70] scales of the BNSS demonstrated high effect sizes, suggesting a stronger reduction of negative symptoms in patients receiving PSM. When comparing NAC vs PLC, the avolition scale [*F*(2, 5.75) = 0.31, *P* = .743, *d* = 0.75] of the BNSS was also slightly more reduced in patients receiving placebo than in the treatment group. Additionally, in the PLC group, the WHO-QOL measure indicated improvements for its quality of life [*F*(2, 13.60) = 1.32, *P* = .299, *d* = 0.61] and psychology [*F*(2, 13.70) = 0.334, *P* = .722, *d* = 0.53] scales, both of which demonstrated higher scores in PLC at the last visit than in the NAC group.

## Discussion

In this randomized multicenter trial, we aimed at evaluating the individual and combined effects of pharmacotherapy with NAC and the integrated preventive psychological intervention (IPPI) for the treatment of CHR-P-patients. The primary endpoint was the transition to psychosis defined as the probability for event-free survival. No significant differences between the treatment groups (IPPI vs PSM/NAC vs PLC) were found.

However, visual inspection of the Kaplan–Meier plot and comparison of survival probabilities indicated that patients receiving NAC (IPPI + NAC: 72.7%, PSM + NAC: 72.7%) showed lower transition rates to psychosis as compared to patients receiving PLC (IPPI + PLC: 56.1%, PSM + PLC: 39.0%). Even though the beneficial effects of NAC are not statistically significant, our findings are in line with the effects of NAC on symptoms in schizophrenia in a recent meta-analysis comparing several anti-inflammatory and antioxidative agents across all stages of schizophrenia.^64^ A meta-analysis by Yolland et al^65^ also showed significantly improved scores on the positive, negative, and total symptom scale of the Positive and Negative Symptom Scale^66^ in patients with schizophrenia receiving NAC. However, even though the overall effects for treatment with NAC might be beneficial, a recent trial comparing NAC and placebo augmentation in clozapine-resistant patients with schizophrenia targeting negative symptoms did not yield significant differences between the groups,^67^ which points to higher efficacy of NAC in early stages of schizophrenia.^68,69^ Nonetheless, to date only a small case series investigated the effects of NAC on CHR-P with mixed results.^28^

### NAC Effects

Comparing the effect size of NAC vs PLC (OR = 0.525) in our study to previous findings in CHR-P patients indicates potentially superior effects compared to a clinical trial that investigated the impact of omega-3 fatty acids on preventing transition to psychosis.^70^ Another study investigated olanzapine as a treatment for CHR-P patients and reported an OR vs control of 0.314,^71^ which is comparable to the effect of NAC in the present study. Thus, considering the advantageous side-effects profile compared to olanzapine, NAC might be a promising treatment for future studies.

In general, previous studies indicate good tolerability of NAC. For example, a study modeling the effects of NAC on neurodegenerative illnesses in various clinical trials found only mild AE, such as gastroesophageal reflux and mild indigestion among patients at dosages between 1800 and 36 000 mg/day.^72^ Similarly, another systematic review reported various smaller side effects of NAC pertaining to different clinical phenotypes.^27,73^ Among these, schizophrenia trials were reporting none or only mild AE. Correspondingly, Miyake et al^30^ did not report serious AE in their case study with CHR-P-patients. In line with these previous findings, our study indicated a similar number of AE in the treatment groups, suggesting good tolerability of NAC among CHR-P patients.

As stated earlier, NAC works as a donator for glutathione (GSH) catalyzing antioxidative and anti-inflammatory effects by modulating glutamate pathways. Low GSH levels in erythrocytes have been shown to predict lower transition rates in individuals with CHR-P.^74^ A remaining question, however, pertains to how fast these NAC-modulated changes can be detected in patients with schizophrenia. In a clinical trial for patients with schizophrenia, a single application of NAC did not alter GSH levels significantly in the medial prefrontal cortex or dorsal anterior cingulate cortex when applying in vivo proton MRS.^75^ Interventions showing good effect sizes for reduction of GSH-levels in patients with schizophrenia were spanning between 2 and 6 months,^24,27^ which is in accord with this study.

### IPPI Effects

Comparing survival probabilities indicated that in patients receiving no active pharmacological compound (PLC), IPPI (IPPI + PLC: 56.1%) was associated with slightly lower transition rates as compared to PSM (PSM + PLC: 39.0%), whereas in patients receiving NAC, there were no differences (IPPI + NAC: 72.7%, PSM + NAC: 72.7%). It is important to note that these results must be interpreted carefully given the small sample size. Nevertheless, in existing research, CBT was often shown to have robust effects on the reduction of transition risk in multiple meta-analyses^76,77^ and is generally recommended for the treatment of CHR-P.^3^ Favorable outcomes of CBT towards preventing transition to psychosis were shown at 12+ months, however, not at 6 months.^78^

In a recent meta-analysis that compared CBT against cognitive remediation therapy and multi-component psychosocial interventions for CHR-P, the latter showed favorable outcomes when looking at measures of social functioning, especially when these therapies exhibited a high degree of manualization.^79^ Even though the present study did not demonstrate improved social functioning as measured with SOFAS and FROGS, we found patients specifically trained in improved perception of emotions with IPPI were presenting with a small tendency towards higher sum-scores in the PFA, which is in accord with existing research.^80^ Future trials with adequate power might additionally be able to demonstrate how the various manualized modules of IPPI^40^ are advantageous to generalized CBT in this regard.

Patients receiving IPPI additionally showed a tendency towards more emotional reactions when faced with stressful events as measured by the BNSS distress scale, which is indicative of reduced negative symptom load.^81^ However, it should be noted that distress did not increase Cronbachs α significantly in confirmatory factor analysis of the BNSS.^81^ Furthermore, IPPI tended to decrease depressive symptoms as measured with CDSS. If this result can be replicated in a larger, more adequately powered trial, IPPI might prove to be beneficial to other psychotherapeutic treatments in this regard. This is due to the fact that many psychosocial interventions did not decrease depressive symptoms when compared to treatment as usual at end of trial or follow-up.^82^ It is important to mention, that the results of all aforementioned secondary analyses, however, did not stay significant after correction for multiple testing. When specifically looking at negative symptoms, both interventions failed to show significant decreases of symptom load in the respective intervention groups. Effect sizes indicate that PLC and PSM groups might possibly be showing higher decreases in the BNSS avolition and alogy scale and higher decreases for the PSM group in the BNSS alogy scale than their respective treatment groups.

### Combined Effects

Even though due to low power we can only take the Kaplan–Meier plot in figure 3 as an indication towards a certain trend of effects, it is interesting that the synergistic effects of NAC and IPPI are similar to those with NAC and PSM. This implies that IPPI might primarily demonstrate effectiveness when used as a supplementary therapy, whereas NAC exhibits efficacy independently. However, it’s important to approach these findings cautiously, as the absence of statistical significance limits interpretation strongly in that regard.

### Drop-out and Transition Rates

Finally, in this study, all groups showed high drop-out rates with two-thirds of all participants dropping out over the course of the study. This warrants attention, as these rates are higher than to be expected in trials with CHR-P patients, that usually present with one-third of participants dropping out over the course of the study.^83^ One reason for high drop-out rates could be the large number of visits during the trial period, as patients sometimes had to appear twice to complete a bi-weekly visit.^84^ As patients did not receive financial compensation, the cost for repeated transportation might have been an issue as well.

Another factor we would like to address is that the total transition rate of all groups after 18 months was higher (34.78%) than in most CHR-P trials, which averages around 20% transitions during the same timespan.^85^ One reason for the higher number of transitions in comparison to other trials might be that the timespan between a first screening and enrollment in the study was rather long. In single cases, it spanned about 6 months when medication had to be tapered off due to strict exclusion criteria. Additionally, because only patients that were showing impairments in social functioning were included in the study,^61^ it is highly likely that these constitute a group that is afflicted by CHR-P more strongly and thus more probable to transition.

### Limitations

The current study has several limitations that warrant attention. Foremost, the present analysis relies on a limited sample size, necessitating a cautious interpretation of all findings within this context.

A reason for the lack of recruitment within this study might be that only a fraction of all patients that were pre-screened went on to participate in the study. Besides not fulfilling inclusion criteria, reasons named for not participating in the trial were: frequent presence of exclusion criteria (in particular due to psychopharmacological treatment), high time requirement for screenings and therapy, commute to the hospital too costly/long or not wanting to participate in either pharmacological or psychotherapeutic study arm. The inclusion criteria in this study were rather restrictive compared to other CHR-P trials. This was due to the fact that criteria were being harmonized along several clinical trials to make comparisons between trials possible. Even though the 2 × 2 design of the trial might be beneficial to investigate the interplay of intervention and compound, future trials should reduce the number of arms and focus on the beneficial effects of NAC or IPPI in isolated studies to reduce the number of participants needed for each study arm.

Another limitation pertains to the exclusion of adolescents <18 years from the study: CHR-P is highly prevalent within this age group and including adolescents might thus have aided in (1) easier recruitment of patients for the study^6^ and (2) enable more integral conclusions about effectiveness of therapies in CHR-P within the generally affected clinical population.

Generally, low recruitment is a problem, frequently encountered by studies with CHR-P patients.^71^ For future clinical trials it might thus be beneficial to allow for longer periods of recruitment to enable meaningful statistical analysis. Conversely, researchers should have a clear idea on how knowledge management and transfer are implemented to preserve recruitment efforts in participating centers when staff is replaced during recruitment periods.

## Conclusion and Future Directions

In conclusion, our study design offered a psychological and pharmacological intervention for CHR-P patients, revealing slightly reduced hazard ratios compared to the corresponding placebo groups. We successfully established the safety and tolerability of NAC in CHR-P patients. Although statistically significant effects of NAC were not observed, the noteworthy effect sizes suggest the potential efficacy and favorable tolerability of NAC as a treatment option for CHR-P patients. This outcome holds promise for guiding future intervention trials.

## Supplementary Material

sgae005_suppl_Supplementary_Tables_S1-S8

## Data Availability

Data can be made available upon request.
