# Supplementary material for: N-Acetylcysteine and a Specialized Preventive Intervention for Individuals at High Risk for Psychosis: A Randomized Double-Blind Multicenter Trial
Source: Schizophr Bull Open. 2024 Feb 28;5(1):sgae005. doi: 10.1093/schizbullopen/sgae005 (PMC11207905; doi:10.1093/schizbullopen/sgae005)
Supplement: sgae005_suppl_Supplementary_Tables_S1-S8 [file sgae005_suppl_Supplementary_Tables_S1-S8.docx]

**Table 1**.: Inclusion and exclusion criteria for the trial. CDSS, Calgary Depression Scale for Schizophrenia; COGDIS, Cognitive Disturbances, GFS, Global Functioning Scale; SIPS, Structured Instrument for Prodromal Symptoms; SPI-A, Schizophrenia Proneness Instrument – Adult Version.

| **Inclusion criteria** | **Exclusion criteria** |
| --- | --- |
| 1. Age 18 – 40 years 2. Subjects with the ability to follow study instructions and likely to attend and complete all required visits. 3. Written informed consent of the subject. 4. Subjects are able to speak, write and understand the German language sufficiently well (at the investigators discretion) to complete all required study procedures. 5. Subjects show impaired social functioning skills as measured by the Global Assessment of Functioning-Scale (Social) (GFS ≤ 7)^a^ 6. Clinical High Risk Criteria    1. Ultra-high risk criteria (Attenuated positive symptoms and/or brief limited psychotic symptoms and/or a combination of familial risk or schizotypal disorder with a significant loss of functioning; severity assessed by the Structured Interview for Prodromal Syndromes, SIPS 5.0) and/or The Basic Symptom Criterion 'Cognitive Disturbances, COGDIS' (2/9 cognitive-perceptive basic symptoms; assessed by the Schizophrenia Proneness Instrument – Adult Version, SPI-A); | **General exclusion criteria:**   1. Known history of hypersensitivity to the investigational drug or to drugs with a similar chemical structure. 2. Simultaneous participation in another clinical trial involving administration of an investigational medicinal product within 30 days prior to clinical trial beginning. The simultaneous participation in a non-interventional clinical trial is permitted in case the subject is nevertheless able and willing to attend and complete all required visits and in case there are no other contraindications ^b^. 3. Subjects with a physical or psychiatric condition which, at the investigator’s discretion, may put the subject at other clinically significant risks than those that are defined as outcome of this study (development of a first psychotic episode, functional deterioration), 4. may confound the trial results or may interfere with the subject’s per protocol participation in this clinical trial. 5. Suicidality in terms of subjects scoring higher than 0 on the CDSS item 8 'Suicidality'. 6. Known substance abuse or dependency within the last month according to DSM-IV-TR.    1. Patients at least have to be in Early Partial Remission in order to participate (Patients have met one or more Substance Abuse or Dependency Diagnosis criteria for at least 1 month but less than 12 months. However, the patient has not met all criteria for a dependence or abuse diagnosis).^a^ 7. Patients with hepatic or renal failure. 8. Patients with known problems of galactose intolerance, clinically significant lactase deficiency or glucose-galactose malabsorption or histamine-intolerance. 9. Subjects with known asthma bronchiale. 10. Subjects with a history of gastrointestinal ulcer. 11. Intake of antitussives (cough-relieving agents). 12. Intake of nitroglycerin. |
|  | **Exclusion criteria regarding special restrictions for females:**   1. Current pregnancy or pregnancy planned within 9 months after start of medication or nursing women. 2. Females of childbearing potential who are not using and not willing to use medically reliable methods of contraception for the entire study duration (such as oral, injectable, or implantable contraceptives, or intrauterine contraceptive devices) unless they are surgically sterilized / hysterectomized or there are any other criteria considered sufficiently reliable by the investigator in individual cases. |
|  | **Indication-specific exclusion criteria:**   1. Having had a psychotic episode lasting longer than 1 week (according to SIPS 5.0). 2. Having symptoms relevant for inclusion potentially arising from a known general medical disorder. 3. Life-time intake of antipsychotic medication with a cumulative dosage of over 30-times the minimum effective dose according to the German treatment guideline for schizophrenia. 4. Intake of antipsychotic medication (independent of duration of intake) within the past 30 days before psychopathological baseline assessments (including self-ratings and screening assessments) at or above the minimum effective dose according to the German treatment guideline for schizophrenia [Exception: quetiapine (see 18.)]. 5. Any intake of antipsychotic medication (i.e., independent of duration of intake) within the past 3 months before psychopathological baseline assessments (including self-ratings and screening assessments) at or above minimum dosage of the '1st episode psychosis' range of German treatment guideline for schizophrenia. (Exception: maximum dosage for aripiprazole 5 mg/d) ^b^. 6. Intake of quetiapine of more than 300 mg (cumulative dosage) or for a duration longer than 7 days within the past month ^a^. 7. Intake of antidepressants during the past 30 days before psychopathological baseline assessments (including self-ratings and screening assessments) ^b^. 8. Intake of benzodiazepines for more than 2 consecutive days during the past 5 days before psychopathological baseline assessments (including self-ratings and screening assessments) ^b^. 9. Intake of mood stabilizers (lithium, valproate, carbamazepine, oxcabazepine, lamotrigine) for more than 30 days (cumulative number of days) during the past three months or any intake during the month before psychopathological baseline assessments (including self-ratings and screening assessments). 10. Any past psychotherapeutic training for prevention purposes specifically targeting impaired social cognition related to psychotic symptoms. |

*Note:*

**^a^**inclusion/exclusion criterion added in later addendums.

^b^inclusion/exclusion criterion discarded in later addendums.

**Table 2.** Outcome-variables and their operationalization.

| **Outcome variable** | **Operationalization** |
| --- | --- |
| **Primary Outcomes:** |  |
| Transition to psychosis / deterioration of symptoms | Structured Interview for Psychosis-Risk Syndromes (SIPS), Schizophrenia Proneness Instrument, Adult version (SPI-A) |
| **Co-primary outcomes:** |  |
| Social Functioning | Social and Occupational Functioning Assessment Scale (SOFAS), Functional Remission of General Schizophrenia (FROGS) |
| **Secondary outcomes:** |  |
| Neurocognition | Digit Symbol Substitution Test (DSST), Verbal Learning and Memory Test (VLMT), Digit Span, Trail Making Test Versions A+B (TMT) |
| Improvement of negative and disorganization symptoms | Brief Negative Symptom Scale (BNSS) and SIPS; remission of CHR-P-criteria |
| Depressive symptoms | Calgary Depression Scale for Schizophrenia (CDSS) |
| Social cognition | Movie for the Assessment of Social Cognition (MASC), the Social Attribution Test Multiple Choice (SAT-MC) and the Pictures of Facial Affect (PFA) |
| Drug safety | Number of AEs and SAEs, laboratory assessments |
| Adherence | Drug Attitude Inventory (DAI), Patient questionnaire on Therapy Expectations and Evaluation (PATHEV) |
| Subjective quality of life | WHO-Quality-of-life questionnaire (WHO-QOL) |

**Table 3.** Demographic data for all treatment groups. IPPI, integrated preventive psychological intervention; MINI, Mini International Neuropsychiatric Interview; NAC, N-acetylcysteine; PSM, psychological stress management; PLC, placebo.

|  |  | **NAC+IPPI (n = 11)** | | **IPPI+PLC (n = 13)** | | **NAC+PSM (n = 11)** | | **PSM+PLC (n = 11)** | | **Total (N = 46)** | |
| --- | --- | --- | --- | --- | --- | --- | --- | --- | --- | --- | --- |
| Variable |  |  | |  | |  | |  | |  | |
| Transitions N(%) |  | 3(28%) | | 5(38%) | | 3(27%) | | 5(45%) | | 16(34%) | |
| Gender (Male/Female) | Count | 8/3 | | 7/6 | | 7/4 | | 6/5 | | 28/18 | |
|  | Column N % | 72.7/27.3 | | 53.8/46.2 | | 63.6/36.4 | | 54.5/45.5 | | 60.9/39.1 | |
| Employment (unemployed) |  | 0 | | 1 | | 3 | | 1 | | 5 | |
| Marital status (single) |  | 8 | | 8 | | 5 | | 7 | | 28 | |
| Urbanization (< 5.000) |  | 0 | | 1 | | 0 | | 5 | | 23 | |
| Urbanization (5.000 – 20.000) |  | 1 | | 0 | | 0 | | 1 | | 2 | |
| Urbanization (20.000 – 100.000) |  | 3 | | 3 | | 2 | | 0 | | 8 | |
| Urbanization (100.000 – 500.000) |  | 3 | | 1 | | 0 | | 3 | | 7 | |
| Urbanization (500.000 – 1.000.000) |  | 0 | | 0 | | 1 | | 1 | | 2 | |
| Urbanization (> 1.000.000) |  | 4 | | 8 | | 8 | | 1 | | 21 | |
| Living situation (alone) |  | 2 | | 2 | | 2 | | 2 | | 8 | |
| Social Connections (min. 1/week) |  | 10 | | 11 | | 8 | | 9 | | 38 | |
| Number of comorbidities per group |  | 10 | | 11 | | 5 | | 11 | | 37 | |
|  |  | Mean | SD | Mean | SD | Mean | SD | Mean | SD | Mean | SD |
| Age (Years) |  | 25.1 | 4.5 | 23.8 | 6.2 | 27.1 | 5.9 | 20.9 | 3.9 | 24.2 | 5.6 |
| Weight (kg) |  | 76.3 | 19.5 | 67.3 | 14.8 | 79.2 | 25.5 | 69.1 | 14.8 | 72.8 | 19.1 |
| Height (cm) |  | 176.8 | 9.8 | 172.8 | 8.2 | 174.2 | 11.3 | 175.5 | 8.9 | 174.8 | 9.4 |
| BMI |  | 24.4 | 5.6 | 22.4 | 3.7 | 25.9 | 7.4 | 22.4 | 5.0 | 23.7 | 5.6 |
| Education (Years) |  | 12.3 | 1.3 | 12.0 | 1.2 | 11.5 | 1.9 | 11.5 | 1.2 | 11.8 | 1.4 |
| Population Density (per sq km) |  | 2112.4 | 1358.3 | 1791.1 | 1461.6 | 2243.8 | 1671.5 | 1518.8 | 1512.1 | 1938.4 | 1472.6 |
|  |  |  |  |  |  |  |  |  |  |  |  |

**Table 4.** Listing of all Adverse Events (AE) within treatment groups. IPPI, integrated preventive psychological

intervention; NAC, N-acetylcysteine; PSM, psychological stress management; PlC, placebo.

|  | IPPI+ACC | IPPI+PLC | PSM+ACC | PSM+PLC | Total |
| --- | --- | --- | --- | --- | --- |
| Abnormal dreams | 2 | 1 | 1 | 3 | 7 |
| Accommodation disorder | 0 | 0 | 0 | 1 | 1 |
| Acute stress disorder | 2 | 0 | 0 | 0 | 2 |
| Advanced sleep phase | 0 | 0 | 1 | 1 | 2 |
| Adverse drug reaction | 0 | 1 | 0 | 0 | 1 |
| Alopecia | 0 | 0 | 0 | 1 | 1 |
| Aptyalism | 0 | 0 | 0 | 1 | 1 |
| Asthenia | 0 | 0 | 0 | 1 | 1 |
| Attention deficit hyperactivity disorder | 1 | 0 | 0 | 0 | 1 |
| Blood bilirubin increased | 2 | 0 | 0 | 0 | 2 |
| Blood cannabinoids increased | 1 | 0 | 0 | 0 | 1 |
| Circulatory collapse | 1 | 0 | 0 | 0 | 1 |
| Constipation | 0 | 1 | 0 | 0 | 1 |
| Cough | 0 | 1 | 0 | 0 | 1 |
| Depressive symptom | 0 | 1 | 0 | 0 | 1 |
| Diarrhoea | 0 | 1 | 0 | 2 | 3 |
| Disease prodromal stage | 0 | 1 | 0 | 0 | 1 |
| Disturbance in attention | 2 | 1 | 1 | 2 | 6 |
| Dizziness postural | 1 | 0 | 0 | 1 | 2 |
| Dry throat | 0 | 0 | 0 | 1 | 1 |
| Dyspepsia | 1 | 0 | 0 | 0 | 1 |
| Fatigue | 0 | 0 | 1 | 2 | 3 |
| Gastrooesophageal reflux disease | 0 | 1 | 0 | 0 | 1 |
| Headache | 2 | 0 | 0 | 0 | 2 |
| Hepatic enzyme increased | 0 | 1 | 2 | 0 | 3 |
| Hyperhidrosis | 1 | 0 | 0 | 0 | 1 |
| Indifference | 0 | 0 | 0 | 1 | 1 |
| Influenza | 1 | 0 | 0 | 0 | 1 |
| Insomnia | 0 | 1 | 0 | 1 | 2 |
| Libido increased | 0 | 0 | 0 | 2 | 2 |
| Lymphopenia | 0 | 1 | 0 | 0 | 1 |
| Memory impairment | 1 | 0 | 1 | 2 | 4 |
| Mental impairment | 0 | 1 | 0 | 0 | 1 |
| Micturition disorder | 0 | 0 | 0 | 1 | 1 |
| Nasopharyngitis | 0 | 0 | 1 | 1 | 2 |
| Nausea | 1 | 1 | 0 | 0 | 2 |
| Norovirus infection | 0 | 1 | 0 | 0 | 1 |
| Oropharyngeal pain | 0 | 1 | 0 | 0 | 1 |
| Palpitations | 1 | 0 | 0 | 1 | 2 |
| Photosensitivity reaction | 1 | 0 | 0 | 0 | 1 |
| Pigmentation disorder | 0 | 1 | 0 | 0 | 1 |
| Pneumonia | 0 | 1 | 0 | 0 | 1 |
| Pruritus | 0 | 1 | 0 | 0 | 1 |
| Rash | 0 | 2 | 0 | 0 | 2 |
| Restlessness | 1 | 2 | 0 | 0 | 3 |
| Saliva altered | 0 | 0 | 0 | 1 | 1 |
| Seasonal allergy | 2 | 0 | 0 | 0 | 2 |
| Sedation | 0 | 1 | 0 | 0 | 1 |
| Skin candida | 0 | 1 | 0 | 0 | 1 |
| Sleep disorder | 1 | 0 | 0 | 1 | 2 |
| Tension | 2 | 1 | 0 | 2 | 5 |
| Tension headache | 0 | 1 | 0 | 0 | 1 |
| Terminal insomnia | 0 | 0 | 1 | 0 | 1 |
| Thinking abnormal | 1 | 0 | 0 | 0 | 1 |
| Urine cannabinoids increased | 1 | 0 | 0 | 0 | 1 |
| White blood cell count decreased | 0 | 1 | 0 | 0 | 1 |
| TOTAL | 29 | 28 | 9 | 29 | 95 |

**Table 5.** Listing of estimated marginal means (M), standard deviation (SD) in the first (V1) and last (V18) visit for IPPI vs PSM. F-values and probabilities are presented for simple main-effects (ME) between groups and interaction (IE) of visit*group within mixed models for co-primary and secondary endpoints. IPPI, integrated preventive psychological intervention; PSM, psychological stress management.

|  | IPPI/PSM | | | | | | | | | | | | | | | | | | | | |
| --- | --- | --- | --- | --- | --- | --- | --- | --- | --- | --- | --- | --- | --- | --- | --- | --- | --- | --- | --- | --- | --- |
| Variable |  | IPPI | | | | | PSM | | | | |  | |  | |  | |  | |  | |
|  |  | N | | M | | SD | N | | M | SD | |  | | F | | df | | *p* | | d^a^ | |
| **Co-primary endpoints** |  |  | |  | |  |  | |  |  | |  | |  | |  | |  | |  | |
| SOFAS Total | V1 | 24 | | 60.13 | | 13.33 | 21 | | 59.48 | 12.18 | | ME group | | .31 | | 1, 28.88 | | .631 | | .07 | |
|  | V18 | 8 | | 78.00 | | 18.49 | 5 | | 78.00 | 5.43 | | IE visit*group | | .23 | | 3, 21.64 | | .872 | |  | |
| FROGS Total | V1 | 24 | | 66.29 | | 8.39 | 20 | | 65.70 | 11.37 | | ME group | | .03 | | 1, 32.02 | | .862 | | .13 | |
|  | V18 | 7 | | 70.86 | | 21.84 | 4 | | 67.50 | 4.51 | | IA visit*group | | .31 | | 3, 16.12 | | .817 | |  | |
| FROGS Daily Life | V1 | 24 | | 18.58 | | 2.95 | 21 | | 17.52 | 3.11 | | ME group | | .00 | | 1, 33.05 | | .960 | | .13 | |
|  | V18 | 7 | | 19.29 | | 6.70 | 4 | | 18.75 | 1.26 | | IA visit*group | | .22 | | 3, 15.49 | | .882 | |  | |
| FROGS Activity | V1 | 24 | | 9.29 | | 2.33 | 21 | | 10.14 | 2.82 | | ME group | | .08 | | 1, 30.22 | | .786 | | .17 | |
|  | V18 | 7 | | 11.43 | | 3.55 | 4 | | 11.00 | 2.16 | | IA visit*group | | .73 | | 3, 19.31 | | .548 | |  | |
| FROGS Relationships | V1 | 24 | | 17.88 | | 2.66 | 20 | | 17.50 | 3.33 | | ME group | | .36 | | 1, 33.65 | | .551 | | .21 | |
|  | V18 | 7 | | 17.71 | | 6.24 | 4 | | 16.50 | 1.29 | | IA visit*group | | .83 | | 3, 16.28 | | .497 | |  | |
| FROGS Quality of adaptation | V1 | 24 | | 10.08 | | 1.69 | 21 | | 9.38 | 2.36 | | ME group | | .00 | | 1, 24.91 | | .952 | | .11 | |
|  | V18 | 7 | | 10.71 | | 2.69 | 4 | | 10.00 | 2.00 | | IA visit*group | | .08 | | 3, 15.56 | | .480 | |  | |
| FROGS Health | V1 | 24 | | 10.46 | | 1.47 | 21 | | 10.81 | 2.75 | | ME group | | .04 | | 1, 27.84 | | .845 | | .04 | |
|  | V18 | 7 | | 11.71 | | 3.25 | 4 | | 11.25 | 1.50 | | IA visit*group | | .13 | | 3, 16.59 | | .941 | |  | |
| **Secondary endpoints** | | |  | |  | | |  | | |  | |  | |  | |  | |  | |  |
| SIPS Positive Scale (last exam) | V1 | 24 | | 6.83 | | 4.25 | 22 | | 8.18 | 3.74 | | ME group | | 1.12 | | 1, 45.59 | | .296 | | .30 | |
|  | V18 | 8 | | 1.38 | | 2.88 | 5 | | .80 | .84 | | IA visit*group | | .67 | | 11, 72.49 | | .760 | |  | |
| SIPS Positive Scale (last week) | V1 | 24 | | 8.79 | | 4.69 | 22 | | 6.32 | 3.77 | | ME group | | 1.34 | | 1, 46.91 | | .253 | | .47 | |
|  | V18 | 8 | | 1.25 | | 2.82 | 5 | | .20 | .45 | | IA visit*group | | 0.99 | | 11, 69.59 | | .469 | |  | |
| SIPS Negative Scale | V2 | 23 | | 7.61 | | 7.08 | 21 | | 6.52 | 5.70 | | ME group | | .06 | | 1, 65.30 | | .813 | | .12 | |
|  | V18 | 8 | | 3.25 | | 4.86 | 5 | | 2.40 | 2.61 | | IA visit*group | | .37 | | 6, 149.44 | | .897 | |  | |
| SIPS Disorganized Scale | V2 | 23 | | 2.87 | | 2.94 | 21 | | 3.57 | 3.33 | | ME group | | 2.45 | | 1, 33.47 | | .127 | | .28 | |
|  | V18 | 8 | | 2.00 | | 3.74 | 5 | | 1.80 | 1.48 | | IA visit*group | | .61 | | 6, 36.42 | | .719 | |  | |
| SIPS General symptoms | V2 | 23 | | 6.30 | | 4.67 | 21 | | 4.67 | 3.80 | | ME group | | .13 | | 1, 47.56 | | .717 | | .12 | |
|  | V18 | 8 | | 1.75 | | 3.11 | 5 | | 1.20 | 1.30 | | IA visit*group | | .81 | | 6, 40.65 | | .568 | |  | |
| BNSS Total | V1 | 23 | | 15.22 | | 13.97 | 21 | | 12.62 | 12.23 | | ME group | | .36 | | 1, 17.64 | | .557 | | .30^a^ | |
|  | V18 | 6 | | 5.33 | | 6.74 | 3 | | 3.67 | 3.51 | | IA visit*group | | .29 | | 2, 12.63 | | .750 | |  | |
| BNSS Anhedonia | V1 | 23 | | 4.65 | | 4.15 | 21 | | 3.81 | 4.35 | | ME group | | .02 | | 1, 12.44 | | .896 | | .17^a^ | |
|  | V18 | 6 | | 1.00 | | 2.00 | 3 | | .67 | 1.15 | | IA visit*group | | .33 | | 2, 8.85 | | .728 | |  | |
| BNSS Reaction | V1 | 23 | | 1.22 | | 1.54 | 21 | | 1.19 | 1.81 | | ME group | | .56 | | 1, 15.05 | | .466 | | .09^a^ | |
|  | V18 | 6 | | .50 | | .55 | 3 | | .67 | .58 | | IA visit*group | | 3.98 | | 2, 9.60 | | .055 | |  | |
| BNSS Social Withdrawal | V1 | 23 | | 3.26 | | 3.37 | 21 | | 2.05 | 2.29 | | ME group | | .33 | | 1, 20.84 | | .573 | | .06^a^ | |
|  | V18 | 6 | | 1.33 | | 1.63 | 3 | | .67 | 1.15 | | IA visit*group | | 3.05 | | 2, 11.38 | | .087 | |  | |
| BNSS Avolition | V1 | 22 | | 2.23 | | 3.13 | 21 | | 2.14 | 2.17 | | ME group | | .42 | | 1, 17.84 | | .527 | | .70^a^ | |
|  | V18 | 6 | | 1.17 | | 1.60 | 3 | | .67 | 1.15 | | IA visit*group | | .89 | | 2, 6.07 | | .457 | |  | |
| BNSS Blunted Affect | V1 | 23 | | 2.43 | | 3.40 | 21 | | 2.00 | 3.10 | | ME group | | .76 | | 1, 18.85 | | .396 | | .22^a^ | |
|  | V18 | 6 | | 1.00 | | 1.55 | 3 | | 1.00 | 1.73 | | IA visit*group | | .28 | | 2, 10,82 | | .760 | |  | |
| BNSS Alogy | V1 | 23 | | 1.52 | | 2.52 | 21 | | 1.43 | 2.60 | | ME group | | .70 | | 1, 15.97 | | .417 | | .55^a^ | |
|  | V18 | 6 | | .33 | | .82 | 3 | | .00 | .00 | | IA visit*group | | .95 | | 2, 13.08 | | .410 | |  | |
| MASC Total | V1 | 23 | | 34.91 | | 4.85 | 21 | | 35.19 | 4.17 | | ME group | | 1.74 | | 1, 17.53 | | .204 | | .43^a^ | |
|  | V18 | 7 | | 39.14 | | 3.13 | 4 | | 34.00 | 4.55 | | IA visit*group | | .25 | | 2, 17.53 | | .784 | |  | |
| MASC Error ToM plus | V1 | 23 | | 4.26 | | 2.09 | 21 | | 5.33 | 3.51 | | ME group | | .42 | | 1, 21.06 | | .525 | | .31^a^ | |
|  | V18 | 7 | | 1.86 | | 1.35 | 4 | | 4.75 | 3.77 | | IA visit*group | | .66 | | 2, 13.21 | | .532 | |  | |
| MASC Error ToM minus | V1 | 23 | | 3.91 | | 1.28 | 21 | | 3.71 | 2.19 | | ME group | | 1.47 | | 1, 20.69 | | .238 | | .23^a^ | |
|  | V18 | 7 | | 2.71 | | 1.25 | 4 | | 5.00 | 4.69 | | IA visit*group | | .66 | | 2, 16.00 | | .529 | |  | |
| MASC Error no mentalizing | V1 | 23 | | 1.91 | | 2.81 | 21 | | .76 | 1.00 | | ME group | | .04 | | 1, 20.00 | | .836 | | .27^a^ | |
|  | V18 | 7 | | .57 | | 1.13 | 4 | | 1.25 | 1.26 | | IA visit*group | | 1.31 | | 2, 17.60 | | .295 | |  | |
| MASC control items | V1 | 22 | | 4.45 | | 1.34 | 21 | | 4.29 | 1.79 | | ME group | | .14 | | 1, 13.01 | | .716 | | .21^a^ | |
|  | V18 | 7 | | 5.14 | | .69 | 4 | | 5.25 | 1.50 | | IA visit*group | | .40 | | 2, 21.59 | | .679 | |  | |
| SAT-MC I | V1 | 20 | | 14.85 | | 2.32 | 20 | | 14.60 | 2.11 | | ME group | | .71 | | 1, 9.00 | | .423 | | .45^b^ | |
|  | V16 | 9 | | 13.44 | | 3.13 | 4 | | 14.00 | 3.56 | | IA visit*group | | - | | - | | - | |  | |
| SAT-MC II | V13 | 17 | | 12.29 | | 4.13 | 7 | | 14.14 | 2.67 | | ME group | | .03 | | 1, 19.63 | | .869 | | .63^b,e^ | |
|  | V18 | 7 | | 13.29 | | 3.04 | 4 | | 14.50 | 2.38 | | IA visit*group | | .52 | | 2, 23.92 | | .476 | |  | |
| PFA | V1 | 23 | | 20.74 | | 2.91 | 21 | | 21.00 | 3.22 | | ME group | | 4.18 | | 1, 16.56 | | .057 | | .82^a^ | |
|  | V18 | 7 | | 23.86 | | 2.85 | 5 | | 20.80 | 2.95 | | IA visit*group | | .25 | | 2, 11.84 | | .780 | |  | |
| WHO-QOL Quality of Life | V1 | 22 | | 3.09 | | .81 | 16 | | 3.25 | .86 | | ME group | | .08 | | 1, 23.28 | | .774 | | .07^a^ | |
|  | V18 | 8 | | 3.37 | | 1.30 | 4 | | 3.75 | .50 | | IA visit*group | | .05 | | 2, 13.64 | | .169 | |  | |
| WHO-QOL Health | V1 | 22 | | 23.95 | | 4.26 | 16 | | 22.75 | 4.12 | | ME group | | 3.06 | | 1, 23.99 | | .093 | | .45^a^ | |
|  | V18 | 8 | | 27.00 | | 6.26 | 4 | | 27.25 | 1.71 | | IA visit*group | | 1.41 | | 2, 22.29 | | .266 | |  | |
| WHO-QOL Psychology | V1 | 22 | | 17.41 | | 3.35 | 16 | | 17.00 | 4.29 | | ME group | | .10 | | 1, 22.78 | | .756 | | .24^a^ | |
|  | V18 | 8 | | 21.00 | | 7.01 | 4 | | 20.75 | 3.86 | | IA visit*group | | .84 | | 2, 14.46 | | .452 | |  | |
| WHO-QOL Relationships | V1 | 22 | | 9.41 | | 3.22 | 16 | | 8.94 | 3.07 | | ME group | | .13 | | 1, 18.42 | | .721 | | .39^a^ | |
|  | V18 | 8 | | 9.38 | | 2.88 | 4 | | 10.00 | 3.37 | | IA visit*group | | 2.54 | | 2, 15.78 | | .111 | |  | |
| WHO-QOL Environment | V1 | 22 | | 30.64 | | 3.58 | 16 | | 28.25 | 4.84 | | ME group | | 5.04 | | 1, 23.68 | | .034 | | .26^a^ | |
|  | V18 | 8 | | 31.75 | | 5.44 | 4 | | 30.25 | 3.30 | | IA visit*group | | 3.56 | | 2, 17,88 | | .050 | |  | |
| WHO-QOL Total | V1 | 22 | | 87.09 | | 12.38 | 16 | | 83.06 | 11.79 | | ME group | | 1.13 | | 1, 19.88 | | .300 | | .11^a^ | |
|  | V18 | 8 | | 95.13 | | 19.04 | 4 | | 95.50 | 7.94 | | IA visit*group | | 2.18 | | 2, 15.19 | | .147 | |  | |
| CDSS Item 1 Depression | V1 | 24 | | .83 | | .70 | 22 | | .64 | .90 | | ME group | | .97 | | 1, 98.93 | | .328 | | .21 | |
|  | V18 | 7 | | .29 | | .49 | 5 | | .20 | .45 | | IA visit*group | | .93 | | 1, 87.61 | | .336 | |  | |
| CDSS Item 2 Hopelessness | V1 | 24 | | .54 | | .59 | 22 | | .41 | .67 | | ME group | | .52 | | 1, 84.65 | | .471 | | .43^d^ | |
|  | V18 | 7 | | .00 | | .00 | 5 | | .00 | .00 | | IA visit*group | | .09 | | 1, 84.33 | | .765 | |  | |
| CDSS Item 8 Suicidality^e^ | V1 | 24 | | .13 | | .34 | 22 | | .05 | .21 | | ME group | | 2.13 | | 1, 101.34 | | .147 | | .54^d^ | |
|  | V18 | 7 | | .00 | | .00 | 5 | | .00 | .00 | | IA visit*group | | .79 | | 16, 305.08 | | .695 | |  | |
| CDSS Item 9 Observed Depr. | V1 | 24 | | .46 | | .66 | 22 | | .09 | .29 | | ME group | | .84 | | 1, 59.01 | | .364 | | .73^d^ | |
|  | V18 | 7 | | .00 | | .00 | 5 | | .00 | .00 | | IA visit*group | | .61 | | 1, 82.85 | | .439 | |  | |
| CDSS Total | V1 | 24 | | 2.08 | | 1.74 | 21 | | 1.33 | 1.74 | | ME group | | .87 | | 1, 103.01 | | .353 | | .09 | |
|  | V18 | 7 | | .57 | | 1.13 | 5 | | .40 | .89 | | IA visit*group | | 3.43 | | 1, 95.28 | | .067 | |  | |
| DAI | V1 | 13 | | 3.62 | | 1.45 | 10 | | 5.20 | 2.44 | | ME group | | .00 | | 1, 22.72 | | .996 | | .15^c^ | |
|  | V13 | 12 | | 4.67 | | 2.27 | 7 | | 5.71 | 3.59 | | IA visit*group | | .47 | | 1, 15.63 | | .502 | |  | |
| PATHEV Hopefulness | V1 | 21 | | 14.86 | | 3.28 | 15 | | 13.67 | 3.70 | | ME group | | .26 | | 1, 40.15 | | .613 | | .17^c^ | |
|  | V13 | 14 | | 15.14 | | 2.66 | 6 | | 15.00 | 2.76 | | IA visit*group + | | .48 | | 2, 30.14 | | .618 | |  | |
| PATHEV Fear | V1 | 20 | | 5.25 | | 2.43 | 15 | | 7.47 | 3.25 | | ME group | | .03 | | 1, 33.69 | | .859 | | .13^c^ | |
|  | V13 | 14 | | 4.79 | | 2.22 | 6 | | 6.00 | 4.05 | | IA visit*group | | .31 | | 2, 26.30 | | .739 | |  | |
| PATHEV Fit | V1 | 19 | | 13.58 | | 3.85 | 15 | | 13.73 | 3.26 | | ME group | | .12 | | 1, 33.85 | | .733 | | .47^c^ | |
|  | V13 | 14 | | 13.50 | | 2.77 | 6 | | 16.00 | 1.41 | | IA visit*group | | 2.34 | | 2, 26.16 | | .115 | |  | |
| PATHEV Total | V1 | 19 | | 33.79 | | 6.17 | 15 | | 34.87 | 6.58 | | ME group | | .23 | | 1, 40.88 | | .635 | | .37^c^ | |
|  | V13 | 14 | | 33.43 | | 4.69 | 6 | | 37.00 | 3.58 | | IA visit*group | | 2.44 | | 2, 32.46 | | .103 | |  | |

*Note:*

^a^only V13 and V18; interpretation of Cohen’s d (Cohen, 1988): small (d = 0.2), medium (d = 0.5), and large (d = 0.8)

^b^only V16

^c^only V08 and V13

^d^no data for V18 due to floor effects

^e^Autoregressive model (AR1) was applied, as autoregressive model with heterogenous variances (ARH1) did not converge.

**Table 6.** Listing of mean (M), standard deviation (SD) in the first (V1) and last (V18) visit for NAC vs. PLC. F-values and probabilities are presented for simple main-effects (ME) between groups and interaction (IE) of visit*group within mixed models for co-primary and secondary endpoints. NAC, N-acetylcysteine; PlC, placebo.

|  | NAC/PLC | | | | | | | | | | | | | | | | | | | |  | |
| --- | --- | --- | --- | --- | --- | --- | --- | --- | --- | --- | --- | --- | --- | --- | --- | --- | --- | --- | --- | --- | --- | --- |
| Variable |  | NAC | | | | | PLC | | | | |  | |  | |  | |  | | |  | |
|  |  | N | | M | | SD | N | M | | SD | |  | | F | | df | | *p* | | | d | |
| **Co-primary endpoints** |  |  | |  | |  |  |  | |  | |  | |  | |  | |  | | |  | |
| SOFAS Total | V1 | 22 | | 58.55 | | 12.75 | 23 | 61.04 | | 12.74 | | ME group | | .04 | | 1, 29.31 | | .747 | | | .22 | |
|  | V18 | 7 | | 71.71 | | 16.95 | 6 | 85.33 | | 6.15 | | IE visit*group | | .70 | | 3, 21.73 | | .564 | | |  | |
| FROGS Total | V1 | 22 | | 64.95 | | 11.34 | 22 | 67.09 | | 7.96 | | ME group | | .41 | | 1, 32.57 | | .529 | | | .18 | |
|  | V18 | 5 | | 70.00 | | 9.87 | 6 | 69.33 | | 22.63 | | IA visit*group | | .58 | | 3, 15.84 | | .636 | | |  | |
| FROGS Daily Life | V1 | 22 | | 18.14 | | 3.18 | 23 | 18.04 | | 2.96 | | ME group | | .36 | | 1, 30.44 | | .553 | | | .24 | |
|  | V18 | 5 | | 20.40 | | 2.61 | 6 | 18.00 | | 6.81 | | IA visit*group | | .05 | | 3, 15.03 | | .984 | | |  | |
| FROGS Activity | V1 | 22 | | 9.36 | | 3.03 | 23 | 10.00 | | 2.07 | | ME group | | .28 | | 1, 31.65 | | .598 | | | .32 | |
|  | V18 | 5 | | 10.40 | | 2.30 | 6 | 12.00 | | 3.52 | | IA visit*group | | 1.15 | | 3, 18.97 | | .353 | | |  | |
| FROGS Relationships | V1 | 22 | | 17.36 | | 3.09 | 23 | 18.05 | | 2.84 | | ME group | | .00 | | 1, 31.73 | | .949 | | | .16 | |
|  | V18 | 5 | | 17.20 | | 2.77 | 6 | 17.33 | | 6.50 | | IA visit*group | | .54 | | 3, 15.88 | | .660 | | |  | |
| FROGS Quality of adaptation | V1 | 22 | | 9.50 | | 2.32 | 23 | 10.00 | | 1.73 | | ME group | | .76 | | 1, 27.06 | | .383 | | | .37 | |
|  | V18 | 5 | | 10.20 | | 1.92 | 6 | 10.67 | | 2.88 | | IA visit*group | | 1.50 | | 3, 15.24 | | .256 | | |  | |
| FROGS Health | V1 | 22 | | 10.59 | | 2.26 | 23 | 10.65 | | 2.08 | | ME group | | .23 | | 1, 32.52 | | .636 | | | .25 | |
|  | V18 | 5 | | 11.80 | | 2.17 | 6 | 11.33 | | 3.20 | | IA visit*group | | .84 | | 3, 16.47 | | .489 | | |  | |
| **Secondary endpoints** | | |  | |  | | | |  | |  | |  | |  | |  | |  |  | |  |
| SIPS Positive Scale (last exam) | V1 | 22 | | 6.32 | | 3.97 | 24 | 8.54 | | 3.86 | | ME group | | .00 | | 1, 44.74 | | .971 | | | .23 | |
|  | V18 | 8 | | 1.38 | | 2.88 | 5 | .80 | | .84 | | IA visit*group | | 1.17 | | 11, 72.05 | | .327 | | |  | |
| SIPS Positive Scale (last week) | V1 | 22 | | 5.05 | | 3.84 | 24 | 8.13 | | 4.16 | | ME group | | .08 | | 1, 46.24 | | .776 | | | .25 | |
|  | V18 | 7 | | 1.14 | | 3.02 | 6 | .50 | | .84 | | IA visit*group | | 1.17 | | 11, 69.58 | | .323 | | |  | |
| SIPS Negative Scale | V2 | 21 | | 7.62 | | 7.30 | 23 | 6.561 | | 5.59 | | ME group | | .16 | | 1, 65.41 | | .692 | | | .12 | |
|  | V18 | 7 | | 4.43 | | 4.69 | 6 | 1.17 | | 2.40 | | IA visit*group | | .142 | | 6, 146.77 | | .990 | | |  | |
| SIPS Disorganized Scale | V2 | 21 | | 3.33 | | 2.96 | 23 | 3.09 | | 3.32 | | ME group | | .13 | | 1, 34.10 | | .719 | | | .35 | |
|  | V18 | 7 | | 2.71 | | 3.77 | 6 | 1.00 | | 1.55 | | IA visit*group | | 3.35 | | 6, 36.44 | | .341 | | |  | |
| SIPS General symptoms | V2 | 21 | | 5.95 | | 3.92 | 23 | 5.13 | | 4.12 | | ME group | | .25 | | 1, 47.69 | | .618 | | | .27 | |
|  | V18 | 7 | | 2.29 | | 3.15 | 6 | .67 | | 1.21 | | IA visit*group | | .75 | | 6, 41.29 | | .616 | | |  | |
| BNSS Total | V1 | 22 | | 16.14 | | 14.84 | 22 | 11.82 | | 10.98 | | ME group | | .00 | | 1, 19.93 | | .981 | | | .02^a^ | |
|  | V18 | 5 | | 5.80 | | 6.83 | 4 | 3.50 | | 4.43 | | IA visit*group | | .01 | | 2, 11.75 | | .986 | | |  | |
| BNSS Anhedonia | V1 | 22 | | 4.32 | | 4.57 | 22 | 4.18 | | 3.95 | | ME group | | .02 | | 1, 12.71 | | .899 | | | .20^a^ | |
|  | V18 | 5 | | 1.40 | | 2.19 | 4 | .25 | | .50 | | IA visit*group | | .55 | | 2, 8.16 | | .598 | | |  | |
| BNSS Reaction | V1 | 22 | | 1.82 | | 1.97 | 22 | .59 | | .96 | | ME group | | .04 | | 1,18.11 | | .841 | | | .15^a^ | |
|  | V18 | 5 | | .60 | | .55 | 4 | .50 | | .58 | | IA visit*group | | .27 | | 2, 8.87 | |  | | |  | |
| BNSS Social Withdrawal | V1 | 22 | | 3.50 | | 3.49 | 22 | 1.86 | | 2.03 | | ME group | | .03 | | 1, 23.59 | | .873 | | | .05^a^ | |
|  | V18 | 5 | | 1.20 | | 1.79 | 4 | 1.00 | | 1.15 | | IA visit*group | | .42 | | 2, 10.45 | | .664 | | |  | |
| BNSS Avolition | V1 | 21 | | 2.43 | | 3.20 | 22 | 1.95 | | 2.10 | | ME group | | 1.42 | | 1, 18.91 | | .249 | | | .75^a^ | |
|  | V18 | 5 | | 1.40 | | 1.67 | 4 | .50 | | 1.00 | | IA visit*group | | .31 | | 2, 5.75 | | .743 | | |  | |
| BNSS Blunted Affect | V1 | 22 | | 2.45 | | 3.60 | 22 | 2.00 | | 2.88 | | ME group | | .65 | | 1, 21.18 | | .428 | | | .29^a^ | |
|  | V18 | 5 | | 1.20 | | 1.64 | 4 | .75 | | 1.50 | | IA visit*group | | .56 | | 2, 9.85 | | .589 | | |  | |
| BNSS Alogy | V1 | 22 | | 1.73 | | 2.86 | 22 | 1.23 | | 2.18 | | ME group | | 1.14 | | 1, 16.28 | | .302 | | | .29^a^ | |
|  | V18 | 5 | | .00 | | .00 | 3 | .50 | | 1.00 | | IA visit*group | | .62 | | 2, 12.63 | | .555 | | |  | |
| MASC Total | V1 | 21 | | 35.48 | | 5.42 | 23 | 34.65 | | 3.51 | | ME group | | .97 | | 1, 16.71 | | .338 | | | .36^a^ | |
|  | V18 | 6 | | 36.83 | | 3.31 | 5 | 37.80 | | 5.72 | | IA visit*group | | .47 | | 2, 17,17 | | .632 | | |  | |
| MASC Error ToM plus | V1 | 21 | | 4.33 | | 2.46 | 23 | 5.17 | | 3.21 | | ME group | | .13 | | 1, 20.28 | | .719 | | | .23^a^ | |
|  | V18 | 6 | | 3.33 | | 3.56 | 5 | 2.40 | | 1.52 | | IA visit*group | | 1.06 | | 2, 13.50 | | .375 | | |  | |
| MASC Error ToM minus | V1 | 21 | | 3.62 | | 1.75 | 23 | 4.00 | | 1.78 | | ME group | | 1.73 | | 1, 21.85 | | .202 | | | .35^a^ | |
|  | V18 | 6 | | 3.17 | | 2.48 | 5 | 4.00 | | 3.74 | | IA visit*group | | .01 | | 2, 14.68 | | .987 | | |  | |
| MASC Error no mentalizing | V1 | 21 | | 1.57 | | 2.93 | 23 | 1.17 | | 1.27 | | ME group | | .04 | | 1, 20.46 | | .845 | | | .38^a^ | |
|  | V18 | 6 | | .83 | | 1.17 | 5 | .80 | | 1.30 | | IA visit*group | | 1.66 | | 2, 17.83 | | .219 | | |  | |
| MASC control items | V1 | 11 | | 4.27 | | 2.00 | 13 | 4.92 | | 1.66 | | ME group | | .08 | | 1, 20.61 | | .780 | | | .36^a^ | |
|  | V18 | 6 | | 5.17 | | 1.17 | 5 | 5.20 | | .84 | | IA visit*group | | 1.01 | | 2, 20.93 | | .381 | | |  | |
| SAT-MC I | V1 | 20 | | 14.50 | | 1.88 | 20 | 14.95 | | 2.50 | | ME group | | 6.64 | | 1, 9.00 | | .030 | | | 1.83^b^ | |
|  | V16 | 5 | | 11.20 | | 2.59 | 8 | 15.13 | | 2.47 | | IA visit*group | | - | | - | | - | | |  | |
| SAT-MC II | V13 | 11 | | 12.36 | | 3.85 | 13 | 13.23 | | 3.88 | | ME group | | .00 | | 1, .13 | | .994 | | | .19^a^ | |
|  | V18 | 5 | | 12.20 | | 2.86 | 6 | 15.00 | | 2.10 | | IA visit*group | | .00 | | 1, .00 | | .994 | | |  | |
| PFA | V1 | 21 | | 20.29 | | 1.95 | 23 | 21.39 | | 3.73 | | ME group | | .39 | | 1, 15.30 | | .542 | | | .17^a^ | |
|  | V18 | 6 | | 21.67 | | 3.67 | 6 | 23.50 | | 2.59 | | IA visit*group | | .20 | | 2, 11.64 | | .818 | | |  | |
| WHO-QOL Quality of Life | V1 | 19 | | 2.89 | | .74 | 19 | 3.42 | | .84 | | ME group | | 3.76 | | 1, 22.69 | | .065 | | | .61^a^ | |
|  | V18 | 6 | | 2.83 | | 1.17 | 6 | 4.17 | | .41 | | IA visit*group | | 1.32 | | 2, 13.60 | | .299 | | |  | |
| WHO-QOL Health | V1 | 19 | | 21.95 | | 4.36 | 19 | 24.95 | | 3.50 | | ME group | | 7.59 | | 1, 18.92 | | .013 | | | .28^a^ | |
|  | V18 | 6 | | 21.17 | | 5.56 | 6 | 30.00 | | 2.28 | | IA visit*group | | 2.50 | | 2, 21.99 | | .105 | | |  | |
| WHO-QOL Psychology | V1 | 19 | | 16.16 | | 3.70 | 19 | 18.32 | | 3.51 | | ME group | | 1.03 | | 1, 22.14 | | .322 | | | .53^a^ | |
|  | V18 | 6 | | 17.17 | | 6.08 | 6 | 24.67 | | 2.66 | | IA visit*group | | .334 | | 2, 13.70 | | .722 | | |  | |
| WHO-QOL Relationships | V1 | 19 | | 8.84 | | 3.29 | 19 | 9.58 | | 2.99 | | ME group | | .71 | | 1,17.56 | | .410 | | | .26^a^ | |
|  | V18 | 6 | | 9.00 | | 3.95 | 6 | 10.17 | | 1.47 | | IA visit*group | | .10 | | 2, 15.69 | | .907 | | |  | |
| WHO-QOL Environment | V1 | 19 | | 28.95 | | 3.61 | 19 | 30.32 | | 4.83 | | ME group | | 3.19 | | 1, 21.09 | | .088 | | | .42^a^ | |
|  | V18 | 6 | | 28.67 | | 4.18 | 6 | 33.83 | | 3.97 | | IA visit*group | | .30 | | 2, 15.49 | | .743 | | |  | |
| WHO-QOL Total | V1 | 19 | | 81.11 | | 11.30 | 19 | 89.68 | | 11.68 | | ME group | | 1.02 | | 1, 21.27 | | .325 | | | .28^a^ | |
|  | V18 | 6 | | 85.33 | | 16.01 | 6 | 105.17 | | 7.31 | | IA visit*group | | 1.09 | | 2, 13.23 | | .365 | | |  | |
| CDSS Item 1 Depression | V1 | 22 | | .68 | | .78 | 24 | .79 | | .83 | | ME group | | .32 | | 1, 97.79 | | .575 | | | .42 | |
|  | V18 | 6 | | .33 | | .52 | 6 | .17 | | .41 | | IA visit*group | | .01 | | 1, 90.56 | | .943 | | |  | |
| CDSS Item 2 Hopelessness | V1 | 22 | | .59 | | .73 | 24 | .38 | | .49 | | ME group | | .04 | | 1, 84.01 | | .837 | | | .52^d^ | |
|  | V18 | 6 | | .00 | | .00 | 6 | .00 | | .00 | | IA visit*group | | 2.18 | | 1, 87.01 | | .143 | | |  | |
| CDSS Item 8 Suicidality^e^ | V1 | 22 | | .09 | | .29 | 24 | .08 | | .28 | | ME group | | 1.83 | | 1, 99.60 | | .179 | | | .19^d^ | |
|  | V18 | 6 | | .00 | | .00 | 6 | .00 | | .00 | | IA visit*group | | .51 | | 16, 305.084 | | .94 | | |  | |
| CDSS Item 9 Observed Depr. | V1 | 22 | | .18 | | .39 | 24 | .38 | | .65 | | ME group | | .68 | | 1, 59.05 | | .681 | | | .11^d^ | |
|  | V18 | 6 | | .00 | | .00 | 6 | .00 | | .00 | | IA visit*group | | 1.06 | | 1, 84.17 | | .305 | | |  | |
| CDSS Total | V1 | 22 | | 3.77 | | 4.14 | 24 | 3.92 | | 2.72 | | ME group | | .38 | | 1, 100.76 | | .540 | | | .25 | |
|  | V18 | 6 | | .67 | | 1.21 | 6 | .33 | | .82 | | IA visit*group | | .24 | | 1, 97.81 | | .629 | | |  | |
| DAI | V1 | 12 | | 4.83 | | 2.21 | 11 | 3.73 | | 1.79 | | ME group | | .02 | | 1, 22.30 | | .901 | | | .13^c^ | |
|  | V13 | 9 | | 5.22 | | 2.82 | 10 | 4.90 | | 2.88 | | IA visit*group | | .07 | | 1, 15.96 | | .791 | | |  | |
| PATHEV Hopefulness | V1 | 19 | | 14.37 | | 3.40 | 17 | 14.35 | | 3.62 | | ME group | | 1.26 | | 1, 37.12 | | .269 | | | .30^c^ | |
|  | V13 | 9 | | 14.78 | | 2.99 | 11 | 15.36 | | 2.38 | | IA visit*group + | | 3.54 | | 2, 31.46 | | .041 | | |  | |
| PATHEV Fear | V1 | 20 | | 6.15 | | 3.36 | 20 | 5.15 | | 2.11 | | ME group | | .53 | | 1, 31.26 | | .473 | | | .26^c^ | |
|  | V13 | 9 | | 5.11 | | 3.41 | 11 | 5.18 | | 2.44 | | IA visit*group | | .73 | | 2, 27.00 | | .490 | | |  | |
| PATHEV Fit | V1 | 20 | | 14.65 | | 2.37 | 20 | 14.75 | | 3.14 | | ME group | | .02 | | 1, 32.81 | | .893 | | | .06^c^ | |
|  | V13 | 9 | | 14.00 | | 2.60 | 11 | 14.45 | | 2.84 | | IA visit*group | | .04 | | 2, 26.77 | | .966 | | |  | |
| PATHEV Total | V1 | 18 | | 34.22 | | 6.70 | 16 | 34.31 | | 5.99 | | ME group | | .12 | | 1, 38.96 | | .729 | | | .37^c^ | |
|  | V13 | 9 | | 33.89 | | 4.46 | 11 | 35.00 | | 4.88 | | IA visit*group | | .98 | | 2, 33,48 | | .387 | | |  | |

*Note:*

^a^only V13 and V18; interpretation of Cohen’s d (Cohen, 1988): small (d = 0.2), medium (d = 0.5), and large (d = 0.8)

^b^only V16

^c^only V08 and V13

^d^only V13

^e^Autoregressive model (AR1) was applied, as autoregressive model with heterogenous variances (ARH1) did not converge.

**Table 7**.: Clinical high risk profiles that were present at enrollment into the trial. More than one criterion can apply. APS, attenuated psychotic symptoms; BLIPS, brief limited intermittent psychotic symptoms; COGDIS, cognitive disturbances; GFRD, genetic and family risk and deterioration syndrome.

| **COGDIS at inclusion** | **APS in inclusion** | **BLIPS at inclusion** | **GFRD at inclusion** |
| --- | --- | --- | --- |
| 25 | 29 | 2 | 16 |

|  |  | **Dropout** | | **Regular End of Study** | |
| --- | --- | --- | --- | --- | --- |
| Variable |  |  | |  | |
| Gender (Male/Female) | Count | 19/13 | | 11/5 | |
|  | Column N % | 59.4/40.6 | | 68.8/31.3 | |
|  |  |  | |  | |
| Urbanization (< 5.000) |  | 2 | | 4 | |
| Urbanization (5.000 – 20.000) |  | 1 | | 1 | |
| Urbanization (20.000 – 100.000) |  | 3 | | 5 | |
| Urbanization (100.000 – 500.000) |  | 5 | | 2 | |
| Urbanization (500.000 – 1.000.000) |  | 1 | | 1 | |
| Urbanization (> 1.000.000) |  | 18 | | 3 | |
| Living situation (alone) |  | 6 (18.8%) | | 2 (12.5%) | |
| Social Connections (min. 1/week) |  | 24 (75%) | | 14 (87.6%) | |
|  |  | Mean | SD | Mean | SD |
| Age (Years) |  | 24.28 | 5.85 | 23.56 | 4.78 |
| Education (Years) |  | 11.56 | 1.40 | 12.31 | 1.3 |
| Population Density (per sq km) |  | 2582.64 | 1297.31 | 1340.71 | 1250.89 |

**Table 8**.: Detailed information on characteristics of patient drop-outs.

As per a recommendation from a Reviewer, we have added additional information on characteristics of patients that have dropped out of the trial in the table above. Additionally, we calculated differences between group mean/median with t-tests and Mann-Whitney U-tests. Interestingly, we found that urbanization operationalized as population density per square kilometre [t(37) = 2.904, *p* = .006] and population size of the city [Mann-Whitney rank test: dropout (median = 6); no dropout (median = 3) U = 120.50, *p* = .004] differed significantly between both groups, with a higher density and a larger population size being indicative of dropout. Since these analyses are not corrected for multiple testing and are not part of the initial statistical analysis plan, we offer them in the supplement in hopes other researchers find them helpful.

**References:**

Gaebel, W., Hasan, A., & Falkai, P. (Eds.). (2019). *S3-Leitlinie Schizophrenie*. Springer-Verlag.
